# Evaluation of the NanoCHIP^®^ Gastrointestinal Panel (GIP) Test for Simultaneous Detection of Parasitic and Bacterial Enteric Pathogens in Fecal Specimens

**DOI:** 10.1371/journal.pone.0159440

**Published:** 2016-07-22

**Authors:** Shifra Ken Dror, Elsa Pavlotzky, Mira Barak

**Affiliations:** Clinical Microbiology Laboratory, Regional Laboratory Haifa and Western Galilee, Clalit Health Services, Nesher, Israel; Cornell University, UNITED STATES

## Abstract

Infectious gastroenteritis is a global health problem associated with high morbidity and mortality rates. Rapid and accurate diagnosis is crucial to allow appropriate and timely treatment. Current laboratory stool testing has a long turnaround time (TAT) and demands highly qualified personnel and multiple techniques. The need for high throughput and the number of possible enteric pathogens compels the implementation of a molecular approach which uses multiplex technology, without compromising performance requirements. In this work we evaluated the feasibility of the NanoCHIP^®^ Gastrointestinal Panel (GIP) (Savyon Diagnostics, Ashdod, IL), a molecular microarray-based screening test, to be used in the routine workflow of our laboratory, a big outpatient microbiology laboratory. The NanoCHIP^®^ GIP test provides simultaneous detection of nine major enteric bacteria and parasites: *Campylobacter* spp., *Salmonella* spp., *Shigella* spp., *Giardia* sp., *Cryptosporidium* spp., *Entamoeba histolytica*, *Entamoeba dispar*, *Dientamoeba fragilis*, and *Blastocystis* spp. The required high-throughput was obtained by the NanoCHIP^®^ detection system together with the MagNA Pure 96 DNA purification system (Roche Diagnostics Ltd., Switzerland). This combined system has demonstrated a higher sensitivity and detection yield compared to the conventional methods in both, retrospective and prospective samples. The identification of multiple parasites and bacteria in a single test also enabled increased efficiency of detecting mixed infections, as well as reduced hands-on time and work load. In conclusion, the combination of these two automated systems is a proper response to the laboratory needs in terms of improving laboratory workflow, turn-around-time, minimizing human errors and can be efficiently integrated in the routine work of the laboratory.

## Introduction

Infectious gastroenteritis is one of the most common diseases worldwide [1, 2] and the resultant diarrheal diseases constitute a global health problem, being common in developed as well as in developing countries, in hospitals and in the community. The severity of these diseases in humans varies from mild symptoms to life-threatening conditions. Common etiologies of diarrheal infections include viruses, bacteria and parasites [2–5]. It is estimated that one-third of the population in developed countries are affected every year by these diseases while in developing countries diarrheal diseases are ranked as the second most common cause of morbidity and mortality in children [2,6].

As infected persons may exhibit similar or non-specific symptoms, the identification of the specific infective agent requires an accurate reliable and timely diagnosis in order to allow appropriate and timely treatment; improved treatment outcomes; enabling efficient disease management and decreasing healthcare costs [7].

Conventional diagnosis of enteric pathogens is commonly associated with performing selective stool cultures (bacterial and amoeba) and various microscopic techniques, i.e. wet mount, mounts after stool concentration and different staining methods [8–10]. These methods are complex, time consuming and labour intensive [11]. The TAT is relatively long since culturing of stool samples usually requires 2–3 days, in addition to the difficulty to culture some of the microorganisms. Moreover, the microscopic detection requires highly trained personnel, is usually subjective characterized by relatively reduced sensitivity and is highly affected by pathogen morphology variations that may limit diagnosis [12–14].

The molecular approach in this field has tremendously increased in the recent years. Specifically, multiplex PCR-based assays that target groups of bacteria or parasites have been developed [1, 12, 14–21]. Accordingly, the utility of the simultaneous detection of various enteric pathogen combinations in a single test was determined [22–25].

The regional laboratories of Haifa and Western Galilee of Clalit Health Services at Nesher, Israel, provide laboratory services for hospitals and community clinics, servicing around 750,000 outpatients who are attended by more than 1,000 physicians from more than 200 outpatient clinics. Our laboratory receives around 20,000 samples and performs over all approximately 80,000 different clinical tests per day. Among them around 1,500 are microbiological samples while 15% are stool samples.

The objective of the current study was to evaluate the use of a novel automated multiplex PCR system, the NanoCHIP^®^ GIP, a molecular electronic microarray system that is intended to simultaneously identify parasites and bacterial gastrointestinal pathogens in clinical faecal specimens [26]. The NanoCHIP^®^ GIP comprises the following pathogens: *Giardia* sp., *Cryptosporidium* spp., *Entamoeba histolytica*, *Entamoeba dispar*, *Dientamoeba fragilis*, *Blastocystis* spp., *Salmonella* spp., *Shigella* spp. and *Campylobacter* spp. We included these pathogens as they are the main pathogens screened locally according to the prevalence in our population, and in view of the treatable or potential requiring treatment diseases. Other bacteria (i.e. *Vibrio cholera*, *Aeromonas*, etc.) and enteric viruses, such as *Norovirus* and *Adenovirus*, are not screened routinely. Screening for *Rotavirus* has been reduced dramatically since the initiation of immunization.

A plethora of literature deals with the utility of the microarray technology in clinical microbiology [27–30]. The NanoCHIP^®^ technology specifically raised interest due to its unique combination of high sample throughput together with its high multiplexing capabilities, addressing to the needs of a high throughput regional laboratory. This system has already been evaluated in comparison to the Luminex xTAG gastrointestinal panel [26], with comparable performances. However, the aforementioned work did not include *Blastocystis* spp. and *Dientamoeba fragilis* in the panel. The aim of our study was to compare the NanoCHIP^®^ performance to conventional methods (culture, direct and stained microscopy) routinely used in our laboratory, including the *Blastocystis* spp.and *Dientamoeba fragilis*, which were not included at the time of evaluation in any molecular panel.

An emphasis was made during this study on the yield and the workflow aspects of the system. The evaluation was performed with both retrospective and prospective stool samples.

## Methods

### Samples

The study was performed at the regional laboratories of Haifa and Western Galilee of Clalit Health Services, Israel, during November 2013 –April 2014. Prospective and retrospective studies were carried out with remnants of faecal samples from symptomatic patients that were sent to our outpatient regional laboratory. Those samples arrived at the laboratory within 3 hours after collecting in the clinic. The samples for stool bacterial culture were collected and transported with Cary-Blair transport medium (Novamed, Israel) and the samples for parasite examination were collected and transported without any additive. The physicians requested either stool culture or parasites or both. The retrospective study was based on our frozen stool bank and included 161 frozen faecal samples, 118 of which were characterized as positives to one or more of the GI panel pathogens by the conventional methods, and 43 which were found to be negative to any of these pathogens. The prospective study included 94 fresh faecal samples 12 of which were characterized as positive to one or more of the GI panel pathogens by the conventional methods. In the retrospective study, the samples chosen for the parasitic detection were stored at -20°C until analysis, and the samples for the bacterial detection were stored at 4°C or at -20°C if analysis was performed more than 3 days after sample collection. The use of all clinical samples in these studies was according to the approval of the Clalit Health Services Ethics Committee, which accepted the study without a need for informed consent.

### Conventional testing methods

The conventional testing included methods used for routine practice in the microbiology laboratory, according to the guidelines of the Israeli Ministry of Health. Samples were cultured for *Campylobacter* spp., *Salmonella* spp. and *Shigella* spp. according to Garcia [31], using Salmonella-Shigella agar, *Campylobacter* blood agar (Novamed, Israel) and CHROMagar for *Salmonella* after enrichment in Muller-Kauffmann tetrathionate broth (Hy-Labs, Israel). *Blastocystis* spp, *Entamoeba histolytica/dispar* and *Dientamoeba fragilis* were detected by xenic biphasic Locke-egg cultivation [32]. *Giardia* sp. was detected by microscopic examination of a direct saline (wet) mount or after concentration by formalin/ethyl acetate method. Detection of oocysts of *Cryptosporidium* spp. was made by microscopic examination of samples that were concentrated and stained by modified Kinyoun's acid fast stain [33]. Monoclonal ELISA E. HISTOLITCA II kit (Techlab, Blacksburg, VA, USA) was used for distinguishing between *Entamoeba histolytica* and *Entamoeba dispar*.

### DNA Extraction

Genomic DNA was extracted from stool samples using MagNA Pure 96 system (Roche Diagnostics Ltd., Switzerland), a benchtop robotic workstation for automated high-throughput purification of nucleic acids. The extraction and purification were performed according to the manufacturer manual using GuHCL reagent (Roche Diagnostics Ltd., Switzerland) and operator's guide. Following the extraction process, 2.5 μl of each DNA sample was transferred for PCR amplification followed by detection analysis by the NanoCHIP^®^ system. The quality of each DNA extraction was indicated by the results of R16 gene [34] that serves as an internal control in the NanoCHIP^®^ GIP test.

### NanoCHIP^®^ GIP assay

The NanoCHIP^®^ GIP panel is composed of the following pathogens (limits of detection (LODs) are indicated upon availability in brackets): *Giardia* sp. (5x10^3^), *Cryptosporidium* spp., *Entamoeba histolytica* (5x10^3^), *Entamoeba dispar* (5x10^3^), *Dientamoeba fragilis* (5x10^3^), *Blastocystis* spp., *Salmonella* spp. (1x10^5^), *Shigella* spp. (1x10^4^), and *Campylobacter* spp. (1.4x10^4^). The LODs were determined by The Public Health Wales Microbiology Lab (see acknowledgement). It should be noticed that it was decided to include *Entamoeba dispar* in the NanoCHIP^®^ GIP panel even though it is not recognized as a pathogen because it is routinely reported by our laboratory.

The targets utilized by the NanoCHIP GIP are presented in Table 1. Extracted DNA (2.5 μl) was inserted into the NanoCHIP^®^ GIP PCR mix in a 96-well plate. PCR amplification and detection were performed according to Savyon NanoCHIP^®^ GIP Application Note (Gastrointestinal Panel Combi II) using reagents supplied by Savyon Diagnostics. Post amplification PCR plates were transferred directly to the NanoCHIP^®^ 400 instrument for detection. Computerized analysis of results was performed automatically by the NanoCHIP^®^ 400 instrument at the end of the run according to the manufacturer's protocol. Each run included internal positive controls for the DNA extraction efficiency, for the PCR amplification quality, and for the NanoCHIP detection signal levels, as well as a negative control for the detection procedure, according to the manufacturer manual. Interpretation of results was performed according to the manufacturer's Application Note. The system was capable to process 96 samples per run.

**Table 1:** Targets detected by the NanoCHIP^®^ GIP test.

PARASITES		BACTERIA	
Pathogen	Target	Pathogen	Target
*Giardia lamblia*	16S rDNA	*Salmonella enterica*	*invA*
*Cryptosporidium* spp *(C*. *parvum and C*. *hominis*)	*Cowp1*	*Shigella* spp. (*S*. *boydii*, *S*. *sonnei*, *S*. *flexneri*, *S*. *dysenteriae*)	*ipaH*
*Entamoeba histolytica*	18S rRNA	*Campylobacter* spp. (*C*. *jejuni and C*. *coli*)	*mapA*, *asp*
*Dientamoeba fragilis*	16S rDNA		
*Blastocystis* spp.	18S rRNA		

### Analysis of discrepant samples

Cases of discrepancies between the NanoCHIP^®^ GIP and the conventional results were re-tested by qPCR using G-DiaBact, G-DiaFrag, G-DiaPara and G-DiaShig qPCR Kits (Diagenode diagnostics, Belgium), according to the manufacturer instructions. The target pathogens and genes in these qPCR kits were as follows: G-DiaBact—*Salmonella enterica (invA*) and *Campylobacter jejuni* (*mapA*); G-DiaFrag–*Dientamoeba fragilis* (5.8S rRNA), G-DiaPara–*Entamoeba histolytica* (18S rRNA), *Giardia lamblia* (18S rRNA) and *Cryptosporidium parvum* (seqmentA); and G-DiaShig–*Shigella* (*ipaH*). In cases in which qPCR was not available the discrepant samples went through sequencing. Following PCR the resulted amplicons were run on agarose gel, extracted from the gel and subjected to sequencing. The sequencing was carried out using primers (provided by Savyon) that were designed according to the specific gene targets as specified in Table 1.

## Results

### Retrospective study

Table 2 represents the comparative performance of the NanoCHIP^®^ GIP test versus the conventional tests routinely used in our laboratory. Overall, in 161 samples were identified 121 enteropathogens by the conventional methods. Fifty nine additional entero-pathogens were found by the NanoCHIP^®^ GIP while defined as negatives by the conventional tests. Obviously several samples contained more than one pathogenic target. These results, that were considered prior to the discrepant analysis as false positives, included 8 *Salmonella* spp., 6 *Shigella* spp., 1 *Campylobacter* sp., 3 *Giardia* sp., 26 *Dientamoeba fragilis* and 15 *Blastocystis* spp. Discrepancies were observed also in 29 samples that were detected by the conventional methods but not by the NanoCHIP^®^ GIP. The false negative results before discrepant analysis included one *Campylobacter* sp. 16 *Dientamoeba fragilis* and 12 *Blastocystis* spp.

**Table 2:** Retrospective comparative study: performance of the NanoCHIP^®^ GIP test versus the conventional tests used in the lab. TP, True Positives; FP, False Positives; TN, True Negatives; FN, False Negatives. According to statistical analysis (Fisher exact test) the results are in concordance (p<0.0001).

Pathogen	TP	FP	TN	FN	Positive agreement	Negative agreement	Total agreement
*Salmonella* spp.	13	8	140	0	100	95	95
*Shigella* spp.	14	6	141	0	100	96	96
*Campylobacter* spp.	26	1	133	1	96	99	99
*Giardia* sp.	14	3	144	0	100	98	98
*Cryptosporidium* spp.	2	0	159	0	100	100	100
*Entamoeba histolytica*	1	0	160	0	100	100	100
*Entamoeba dispar*	1	0	160	0	100	100	100
*Dientamoeba fragilis*	24	26	95	16	60	77	74
*Blastocystis* spp.	26	15	122	12	68	89	92

The discrepant analysis (Table 3), revealed that 40 out of the aforementioned 59 NanoCHIP^®^ GIP supposed false positive results were true positive. Discrepant analysis of the NanoCHIP^®^ GIP false negative results (namely, not detected by the NanoCHIP^®^ GIP) has demonstrated that 26 out of the 29 samples were indeed true negatives, while 1 *Dientamoeba fragilis* and 2 *Blastcystis* spp. remained undetected in the NanoCHIP^®^ GIP. Overall, following the discrepant analysis it appeared that the NanoCHIP^®^ GIP correctly detected 161 positive results to any of the pathogens in the panel while only 124 positive samples were detected by the conventional tests. When the NanoCHIP^®^ GIP was compared to the combination of the conventional methods and the qPCR/sequencing, the positive agreement for most pathogens was 100% with exception of 98% for *Dientamoeba fragilis and 95%* for *Blastcystis* spp. (Tables 2 and 3). The negative agreement varied between 95 and 100%, as well as the range of the total agreement.

**Table 3:** Discrepant analysis results following the discrepancies shown in Table 2. TP, True Positives; FP, False Positives; TN, True Negatives; FN, False Negatives. According to statistical analysis (Fisher exact test) the results are in concordance (p<0.0001).

Pathogen	TP	FP	TN	FN	Positive agreement	Negative agreement	Total agreement
*Salmonella* spp.	14	7	140	0	100	95	95
*Shigella* spp.	17	3	141	0	100	99	98
*Campylobacter* spp.	27	0	134	0	100	100	100
*Giardia* sp.	16	1	144	0	100	99	99
*Dientamoeba fragilis*	48	2	110	1	98	98	96
*Blastocystis* spp.	35	6	118	2	95	95	95

### Prospective study

Table 4 represents the comparative performance of the NanoCHIP^®^ GIP test versus the conventional methods, in the prospective context. Overall, 94 samples were tested, of which 12 were classified as positive by the conventional methods and 46 were positive to any of the pathogens only by the NanoCHIP^®^ GIP, while negatives by the conventional tests. The NanoCHIP^®^ GIP additional identified 2 *Salmonella* spp., 3 *Shigella* spp., 4 *Campylobacter* spp., 20 *Dientamoeba fragilis* and 17 *Blastocystis* spp. There were no NanoCHIP^®^ GIP false negative results and all positive conventional methods results were in concordance with the NanoCHIP^®^ GIP results. The discrepant analysis revealed that 43 out of the aforementioned 46 samples detected by the NanoCHIP^®^ GIP were true positives (Table 5). Three NanoCHIP^®^ GIP positive samples remained undetected by the reference molecular methods and therefore were considered as NanoCHIP^®^ GIP false positives. These samples included one *Salmonella* spp., one *Shigella* spp. and one *Campylobacter* spp. Following the discrepant analysis it appeared that the NanoCHIP^®^ GIP test detected prospectively 55 samples confirmed as positives to any of the pathogens in the panel while only 12 samples were detected by the conventional tests. When the NanoCHIP^®^ GIP was compared prospectively to the combination of the conventional methods and the qPCR/sequencing, the positive agreement for all the pathogens was 100% (Tables 4 and 5). The negative agreement increased to 99–100%, as well as the total agreement. The NPV for all the pathogens was 100%, while the PPV was 75%, 83%, 86%, 100%, and 100% for *Salmonella* spp., *Shigella* spp., *Campylobacter* spp., *Dientamoeba fragilis* and *Blastocystis* spp. respectively (Table 5).

**Table 4:** Prospective comparative study: performance of the NanoCHIP^®^ GIP test versus the conventional tests used in the lab. TP, True Positives; FP, False Positives; TN, True Negatives; FN, False Negatives. According to statistical analysis (Fisher exact test) the results are in concordance (p<0.0001).

Pathogen	TP	FP	TN	FN	Positive agreement	Negative agreement	Total agreement
*Salmonella* spp.	2	2	90	0	100	98	98
*Shigella* spp.	3	3	88	0	100	97	97
*Campylobacter* spp.	3	4	87	0	100	96	96
*Dientamoeba fragilis*	2	20	72	0	100	78	79
*Blastocystis* spp.	2	17	75	0	100	81	82

**Table 5:** Discrepant analysis results following the discrepancies shown in Table 4. TP, True Positives; FP, False Positives; TN, True Negatives; FN, False Negatives; PPV, Positive Predictive Value; NPV, Negative Predictive Value.

Pathogen	TP	FP	TN	FN	Positive agreement	Negative agreement	Total agreement	PPV	NPV
*Salmonella* spp.	3	1	90	0	100	99	99	75*	100
*Shigella* spp.	5	1	88	0	100	99	99	83*	100
*Campylobacter* spp.	6	1	87	0	100	99	99	86*	100
*Dientamoeba fragilis*	22	0	72	0	100	100	100	100	100
*Blastocystis* spp.	19	0	75	0	100	100	100	100	100

*not statistically significant. According to statistical analysis (Fisher exact test) the results are in concordance (p<0.0001).

An additional benefit of this evaluation was the detection of the co-infections as detected by the NanoCHIP^®^: Table 6 represents the cases of mixed infections versus the pathogens characterized by the conventional methods. Since those samples were not fully elaborated by the conventional methods, a full comparison was not completed. However, there were no cases of mixed infections that were detected by the conventional methods and not detected by the NanoCHIP^®^ GIP.

**Table 6:** Mixed infections detected by the laboratory using the NanoCHIP^®^ GIP test. n: number of samples.

Test requested by physician	Pathogens detected by conventional methods (n)	Co-pathogens detected by the NanoCHIP^®^ GIP
Entero-bacteria	*Salmonella* spp. (6)	*Campylobacter* spp., *Shigella* spp., *Blastocystis* spp.
Entero-bacteria	*Shigella* spp. (3)	*Campylobacter* spp., *Dientamoeba fragilis*
Entero-parasites	*Giardia* sp. (3)	*Campylobacter* spp., *Dientamoeba fragilis*
Entero-parasites	*Dientamoeba fragilis* (6)	*Campylobacter* spp, *Shigella* spp., *Salmonella* spp., *Giardia* sp.
Entero-parasites	*Blastocystis* spp. (5)	*Campylobacter* spp, *Shigella* spp., *Giardia* sp.

## Discussion and Conclusions

The purpose of the current study was to evaluate the NanoCHIP^®^ GIP test as a molecular-based diagnostic system for routine detection of enteric bacterial and parasitic pathogens in a rather big outpatient laboratory. We decided to include in the panel the main pathogens screened locally according to population, geography conditions and treatable or potential requiring treatment diseases. This evaluation was performed as a part of the efforts to present a valid alternative to the currently used non-molecular detection methods, in view of the well-known advantages of using molecular methodologies for the concomitant detection of different enteric pathogens in human stool, advantages that have been demonstrated in numerous publications [14, 18, 35, 36]. For this purpose two types of studies were performed, a retrospective and prospective study. In both cases the results of the NanoCHIP^®^ GIP were first compared to the routine conventional methods (i.e. culture and microscopic examination) and discrepant results were further tested by qPCR and/or sequencing. The combined results in the retrospective study clearly demonstrated a higher diagnostic yield achieved by the NanoCHIP^®^ GIP in *Salmonella* spp, *Shigella* spp, *Campylobacter* spp, *Giardia* sp., *Dientamoeba fragilis* and *Blastocystis* spp. when compared to the conventional methods. All the samples detected as positive by the conventional methods were detected also by the NanoCHIP^®^ GIP. There were pathogens that were detected only by the NanoCHIP^®^ GIP and therefore these results were defined as false positive, however the negative agreement values remained above 95%. One of the prominent consequences of using this molecular approach was the effective detection of *Blastocystis* spp. and *Dientamoeba fragilis* infections in both, the retrospective as well as the prospective studies. The clinical significance of detecting these two microorganisms is well documented [12, 37–40]. *Dientamoeba fragilis* is in particular difficult to diagnose by microscopic methods due to rapid degradation of its trophozoites, and therefore its detection demonstrates the advantage of using molecular methods for diagnosis of this organism [41]. Yet, it is notable that 3 positive results out of 161 remained undetectable by the NanoCHIP^®^ GIP, while 2 *Dientamoeba fragilis* and 6 *Blastocystis* spp. NanoCHIP^®^ GIP results were defined as false positives. However, the positive and negative agreement values were not substantially reduced, namely above 95% positive and negative agreement values for both pathogens. It should be mentioned that all the false positive results were defined according to the qPCR, however the DNA from those samples was not suitable for further sequencing. Furthermore, the NanoCHIP^®^ GIP enabled the separate detection of 24 *Dientamoeba fragilis* and 9 *Blastocystis* spp., as well as the detection of 19 mixed infections (Table 6) that were not noticed using conventional methods. This fact probably stems from the increased sensitivity of the microarray and it should be noted that not all the samples sent for conventional detection included requests for both bacterial culture and stool parasites. The detection of mixed infections by the NanoCHIP^®^ GIP appeared to be less cumbersome in terms of laboratory work and in the view of the ability to achieve multiple pathogens detection due to the multiplexing capabilities in one assay. This observation is especially notable due to the relatively high prevalence of multi-pathogen appearance in gastrointestinal tract infections [42, 43], and the fact that this phenomenon has potential treatment implications.

The prospective study included only 5 of the most prevalent pathogens since the other 3 pathogens (*Cryptosporidium* spp., *Entamoeba histolytica* and *Entamoeba dispar*), have a lower prevalence as noticed also in the retrospective study. The first comparison with the conventional methods revealed a high rate of positive results detected by the NanoCHIP^®^ GIP but not detected by the conventional methods, with emphasis on *Dientamoeba fragilis* and *Blastocystis* spp. Consequently, while the positive agreement rates were 100% for all the detected pathogens, the negative agreement rates of these two pathogens were substantially reduced. However, the discrepant analysis has shown full agreement of the molecular methods with the NanoCHIP^®^ GIP results for these two pathogens, and for most of the other detected pathogens. The main difference between the retrospective and the prospective studies results was in the negative agreement before the discrepant analysis. It should be taken in consideration the inclusion of more positive samples in the retrospective study as well as the abundance of *Dientamoeba fragilis* and *Blastocystis* spp. in the prospective study. Overall, the advantage of using the NanoCHIP^®^ GIP for detection of the pathogens in the panel was re-assured in the prospective context, making it less prone to subjectivity. It is notable that the NPV values for all the pathogens were 100%, as the basic requirement from a typical screening method like the NanoCHIP^®^ GIP. The PPV for *Salmonella* spp., *Shigella* spp. and *Campylobacter* spp. were reduced and this may be attributed to the overall low number of positive samples detected prospectively; i.e. one false positive for each of the indicated pathogens while 3–6 true positive samples were collected in the frame of this study. Another comprehensive prospective study using the same panel (a poster at ECCMID 2015) comprised higher numbers of *Shigella* spp. - 19 samples and *Campylobacter* spp. -15 samples with similar results. The results presented in the current study emphasize the advantages of utilizing molecular diagnostic methods over the traditional methods, and the NanoCHIP^®^ GIP represents a good paradigm for this widely accepted approach. Conventional diagnostic tests, especially culture and microscopy techniques, necessitate the use of highly trained personnel. Indistinguishable morphologies (e.g. *Entamoeba histolytica* and *Entamoeba dispar*), variations in pathogen morphology, and mixed infections may also account for under-diagnosis or incorrect detection. Overall, it has been shown that in addition to the substantial higher diagnostic yield of the NanoCHIP^®^ GIP, the results of the molecular methods used for microorganisms determination in the discrepant cases were mostly identical to the results of NanoCHIP^®^ GIP results, confirming the general capabilities of this molecular method to overcome many of the conventional methods pitfalls and to provide a better diagnostic outcome [1, 26, 44].

The analytical sensitivity of the NanoCHIP^®^ GIP has been shown to be similar to the sensitivity of other molecular assays for enteropathogens, demonstrating LODs ranging between 10^3^–10^5^ [45–46].

In addition to the performance aspect, the molecular approach represents a significant alleviation of the burden of the routine laboratory work. The NanoCHIP^®^ technology together with the automated DNA extraction system (MagnaPure, Roche) represent an automated relatively high-throughput combined system. The automation of the microarray molecular assays enabled the reduction of the turn-around time, as well as the hands-on-time by omitting the enrichment steps, the use of selective culture media, time-consuming microscopy and the dependency upon the experience and special skills of personnel (especially of the microscopic evaluator), thus allowing for a more objective and clear interpretation of results. However, taking in consideration the need for antimicrobial resistance testing of isolates, cooperation with public health agencies and supporting outbreak investigations, any workflow algorithm using enteric molecular methods should include also culturing the positive bacterial samples and preserving the original stools for pathogen typing (e.g. PFGE or sequence- based typing) if needed.

The composition of the NanoCHIP^®^ GIP panel was designed to respond to the specific needs of this laboratory in terms of the most prevalent bacterial and parasitic pathogens that are detected routinely. The system represents a unique combination between the multiplex detection of all 9 relevant targets together with high sample throughput that is required to be processed on a daily basis. Overall, this combination has proved to work well and to comply with the laboratory's needs. Moreover, the panel combination can be tailored according to different geographical areas and needs. For example, *Norovirus* which is involved in many outbreaks worldwide, is not prevalent in our country, however, according to the manufacturer, inclusion of such a pathogen in this panel can be easily achieved upon request.

A substantial addition to the work load in the lab is the need for concomitant detection of parasites and bacteria which is conventionally carried out in two separate processes. Physicians do not always request detection of both, bacteria and parasites, in all samples and often the requisition includes only the diagnosis of one type of pathogen [1, 14]. The microarray assay eliminates the constraint of different samples, separate analyses of parasites and bacteria and significantly reduces sample-to-result turn-around times from 48–72 hours for the conventional methods to the next day results.

Taking in account all the procedures and media required by the conventional methods, it could be claimed that the cost of the molecular methods is similar to the conventional ones and the simplicity of the molecular procedures makes these assays an essential tool in the modern laboratory. Our assessment revealed that the cost per detected pathogen when using conventional methods (reagents, consumables, overhead and manpower) was approximately $37.5. In comparison, at medium-high throughput settings (i.e., >50 samples/run), the cost of reagents and instrumentation required for the molecular analysis on the NanoCHIP^®^ platform does not exceed $30 per sample, while decreasing dramatically costs associated with labor, laboratory space and waste removal. These calculations demonstrate the ability to adopt this method in our laboratory workflow without additional costs. A comprehensive cost effectiveness evaluation between molecular and conventional methods using different enteric panels, was discussed by Halligan et al. (24). Moreover, the reduction in time to result due to utilising molecular methods can shorten the length of stay of patients in the hospitals and thus reducing healthcare overall costs.

In conclusion, our data indicate that the increased detection yield gained by the microarray assay, its unique combination of target multiplexing capabilities together with its mid-high throughput sample processing and the overall compatibility of the whole system with our laboratory routine workflow, make the NanoCHIP^®^ GIP a valid and an effective tool for routine screening of stool specimens for gastrointestinal pathogens. This molecular system has proven to have a practical added value in terms of broadening detection capabilities, reducing the sample-to-result turn-around-time, and valuable decreasing the laboratory workload.
